# HIF Prolyl Hydroxylase Inhibitors for COVID-19 Treatment: Pros and Cons

**DOI:** 10.3389/fphar.2020.621054

**Published:** 2021-01-29

**Authors:** Andrey A. Poloznikov, Stepan A. Nersisyan, Dmitry M. Hushpulian, Eliot H. Kazakov, Alexander G. Tonevitsky, Sergey V. Kazakov, Valery I. Vechorko, Sergey V. Nikulin, Julia A. Makarova, Irina G. Gazaryan

**Affiliations:** ^1^ Faculty of Biology and Biotechnology, HSE University, Moscow, Russia; ^2^ P. A. Hertsen Moscow Oncology Research Center, Branch of the National Medical Research Radiological Center, Ministry of Health of the Russian Federation, Moscow, Russia; ^3^ School of Biomedicine, Far Eastern Federal University, Vladivostok, Russia; ^4^ Department of Anatomy and Cell Biology, New York Medical College, Valhalla, NY, United States; ^5^ Department of Chemistry and Physical Sciences, Dyson College of Arts and Sciences, Pace University, Pleasantville, NY, United States; ^6^ City Clinical Hospital No 15 Named After O. M. Filatov, Moscow, Russia; ^7^ Chemical Enzymology Department, M. V. Lomonosov Moscow State University, Moscow, Russia

**Keywords:** SARS-CoV, hypoxia inducible factor, roxadustat, vadadustat, adaptaquin, neuradapt

## Abstract

The review analyzes the potential advantages and problems associated with using HIF prolyl hydroxylase inhibitors as a treatment for COVID-19. HIF prolyl hydroxylase inhibitors are known to boost endogenous erythropoietin (Epo) and activate erythropoiesis by stabilizing and activating the hypoxia inducible factor (HIF). Recombinant Epo treatment has anti-inflammatory and healing properties, and thus, very likely, will be beneficial for moderate to severe cases of COVID-19. However, HIF PHD inhibition may have a significantly broader effect, in addition to stimulating the endogenous Epo production. The analysis of HIF target genes reveals that some HIF-targets, such as furin, could play a negative role with respect to viral entry. On the other hand, HIF prolyl hydroxylase inhibitors counteract ferroptosis, the process recently implicated in vessel damage during the later stages of COVID-19. Therefore, HIF prolyl hydroxylase inhibitors may serve as a promising treatment of COVID-19 complications, but they are unlikely to aid in the prevention of the initial stages of infection.

## Introduction

In 2019, a spike in the cases of lethal pneumonia caused by the global spread of the SARS-CoV-2 virus led to an urgent need to develop effective therapies, especially vaccines (Chan et al., 2020; Zhou et al., 2020). The virus belongs to the Coronaviridae family, which consists of 40 enveloped viruses containing single-stranded (+)RNA (Holmes, 2001; Lai and Holmes, 2001). Most of these viruses frequently circulate in human populations, causing non-life threatening intestinal and respiratory infections (Corman et al., 2019). However, some members of the family such as the severe acute respiratory syndrome (SARS-CoV) and the Middle East respiratory syndrome (MERS-CoV) coronaviruses cause severe illnesses (Fehr et al., 2017). The tropism of the SARS-CoV-2 virus, the structure of its receptor binding domain, the virus’s mechanism of entry into the target cell and its life cycle are well documented in (McKee et al., 2020; Sternberg et al., 2020). SARS-CoV infection also disrupts gastrointestinal tract function, as evidenced by the presence of the virus in biopsy and in stool samples even in discharged patients (Leung et al., 2003). SARS-CoV infection leads to the damage of the respiratory system and to thrombosis, and is accompanied by hypoxia which contributes to the severity of disease. Very recently a number of warnings have been reported with respect to CNS damage and long-term side effects in patients who have recovered from COVID-19 (the infectious disease caused by SARS-CoV-2 virus). In this paper we focus on the possibility of using anti-hypoxic drugs under development to prevent, treat, or ease the long-term side effects of COVID-19.

## Ischemia and Hypoxia

The history of ischemic stroke treatment with drugs is discouraging because no drug can enter a lesion deprived of blood supply. The biggest difference between ischemia and hypoxia is that the former involves both oxygen and glucose (and other nutrient) deprivation, so no energy is available to activate the pro-survival genetic programs, whereas hypoxia only involves low oxygen levels. The latter case can be potentially treated with drugs activating the anti-hypoxic response at the cellular level, as the nutrients are still available. Hypoxia without glucose deprivation is exactly what happens with COVID-19. When the virus infects the body, the lung damage results in low oxygen tension/insufficient oxygen in the blood, but as long as the heart is functioning, the blood’s access to the tissues is uncompromized. This peculiar characteristic of COVID-19 leads to a hypothesis stating that activation of the intrinsic anti-hypoxic program non-pharmacologically, with ischemic preconditioning, (Hertzog et al., 2020; Serebrovska et al., 2020), or pharmacologically, with small molecule drugs, can be used to treat COVID-19 as a way to lessen the damage caused by hypoxia**.**

Low oxygen levels trigger a general organismal response due to the inhibition of various oxygen-dependent enzymes. It took more than 2 decades of intense research to fully appreciate the role that these enzymes play in cell fate. These oxygenases are either iron or FAD dependent, and they control the stability of various transcription factors and key proteins relevant to cell division, demethylate DNA and histones, and result in many other effects. During the early 1990’s, the stabilization of a transcription factor, a hypoxia inducible factor (HIF) that switched the cell from aerobic to anaerobic glycolysis, was documented in hypoxia (Wang and Semenza, 1995a). However, only a decade later, a major regulatory enzyme regulating the stability of the HIF protein was identified and described (Bruick and McKnight, 2001). The last 2 decades led to the discovery of many enzymes from the same group performing indispensable functions within the living cell (Wilson et al., 2020).

**FIGURE 1:**
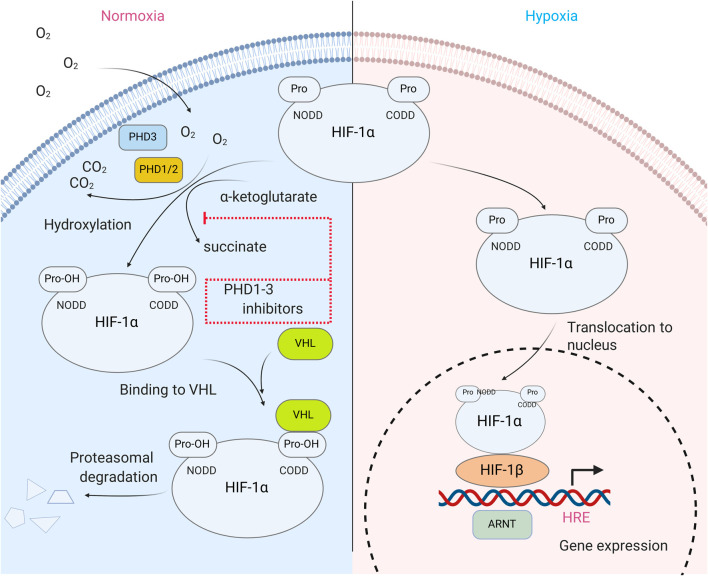
HIF prolyl hydroxylase in HIF stability regulation. In normoxia, HIFα subunit is hydroxylated at Pro 402 and 564 via reaction catalyzed by isoenzymes of HIF prolyl hydroxylase (PHD1-3). Hydroxylated prolines are recognized by VHL protein, a component of ubiquitin ligase complex, and thus, eventually target HIF for degradation. In hypoxia, PHD activity is strongly reduced, which results in HIFα protein stabilization, its dimerization with HIFβ subunit and translocation to the nucleus, where HIF dimer binds on the enhancer sequence of HIF target genes and activates gene transcription.

HIF is the most well-known substrate of HIF prolyl hydroxylase. It activates a group of genes participating in glucose metabolism, control of intracellular pH, angiogenesis, erythropoiesis, mitogenesis, etc., and it is composed of two subunits, an α-subunit that quickly degrades in the presence of oxygen (its half-life is less than 5 min in 21% O_2_) and a stable β-subunit (Wang and Semenza, 1995b; Wang et al., 1995). The HIFα subunit is regulated *via* post-transcriptional modifications such as phosphorylation, acetylation, and hydroxylation. Hydroxylation appears to be the main regulator of HIFα protein stability and is controlled by non-heme iron α-ketoglutarate dependent dioxygenases known as HIF prolyl hydroxylases (HIF PHD). Hydroxylation of HIF-1α, Pro564 and/or 402 is required for its interaction with the von Hippel-Lindau (VHL) tumor suppressor, leading to the formation of a complex that ubiquitinates HIFα and sentences it to subsequent proteosomal degradation (Kaelin, 2005) (Figure 1.). The inhibition of HIF PHD is sufficient to stabilize HIFα, and thus, to activate the HIF-driven genetic program. However, HIF PHD exists in three isoforms which have more than a dozen client substrates in addition to HIF, such as ATF4, p53, biological clock protein HCLK2, and many other important cell fate protein effectors. HIF PHD isoforms have very similar active sites and as a result, the inhibition of HIF PHD activity leads to much more profound effects than to just the stabilization of the HIF protein.

## HIF PHD Inhibitors

**FIGURE 2:**
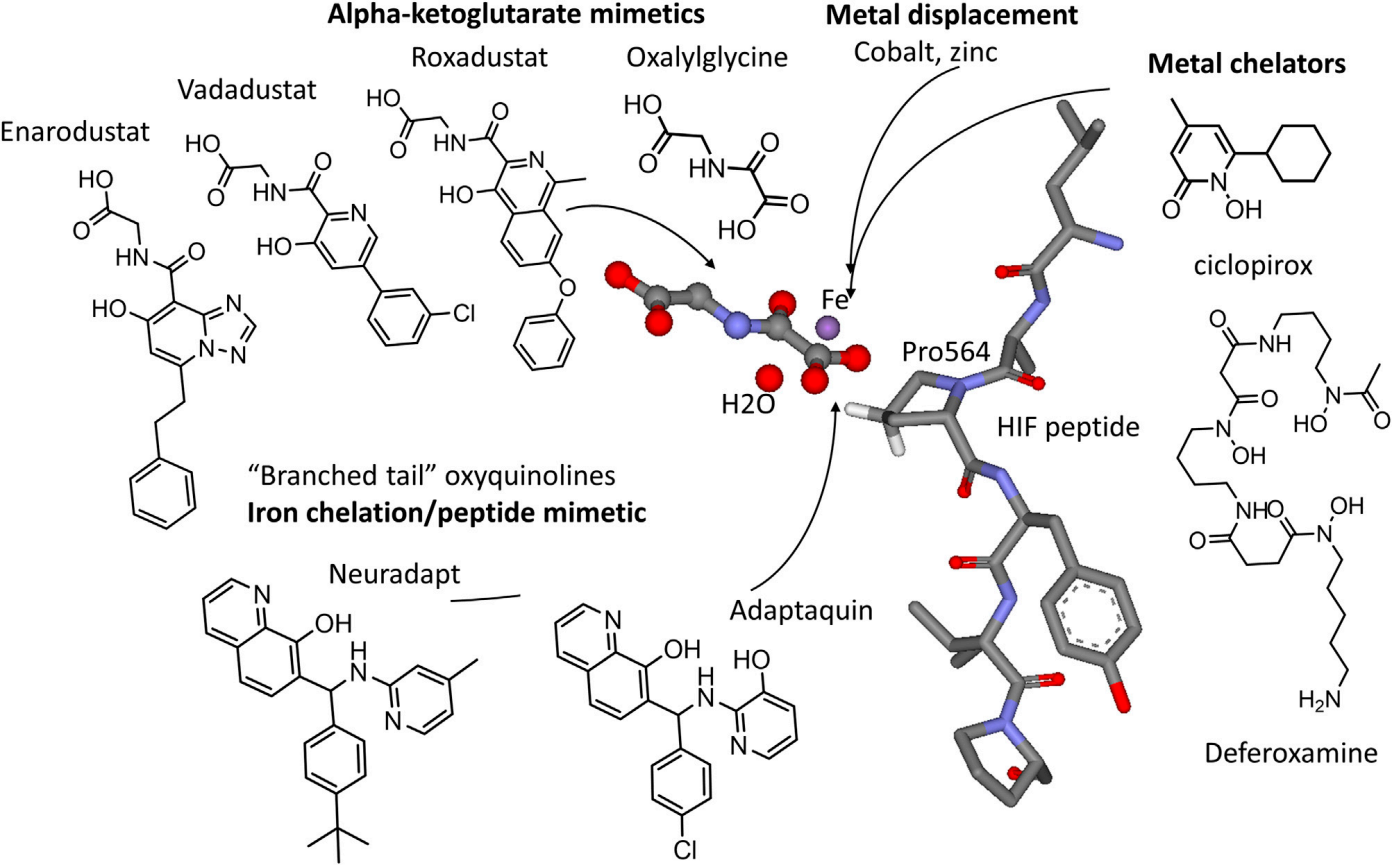
HIF PHD inhibitors and their targets in the active site of HIF prolyl hydroxylase (3HQR.pdb). Alpha-ketoglutarate mimetics include N-oxalylglycine (DMOG), Roxadustat, Vadadustat, and Enarodustat. Metal chelators, e.g., ciclopirox, deferoxamine (DFO), and various transition metals target the active site iron (in purple). Branched tail oxyquinolines (adaptaquin and neuradapt) provide two ligands to chelate iron and to mimic the fold of the HIF peptide.

HIF PHD inhibitors have a great potential for the treatment of anemia of different etiology (Joharapurkar et al., 2018; Kular and Macdougall, 2018) by triggering the production of endogenous erythropoietin and by activating erythropoiesis. For this purpose, HIF PHD inhibitor-based drug formulations contain the active ingredient in a tablet form suitable for oral administration. HIF PHD inhibitors include iron displacing compounds, iron chelators, α-ketoglutarate mimetics, and HIF competitors (Figure 2).

The first group has compounds displacing iron from the active center of HIF-PHD. The direct binding of iron to the active center of HIF-PHD is an essential step for the activation of the enzyme, while the substitution of iron leads to its inactivation. This group includes metal ions such as manganese, zinc, nickel and cobalt. Cobalt, a widely used hypoxia mimic, works by substituting iron in the active center of HIF PHD to inactivate the enzyme and to stabilize HIF, thus, leading to an increased expression of dopamine in the plasma of rats (Louise et al., 2009). However, cobalt is known to be toxic, whereas zinc has been recommended for the treatment of COVID-19 (Alexander et al., 2020) even though the mechanism of zinc’s inhibitory effect on viral replication is not known.

The second group includes iron chelators. Deferoxamine (DFO), a bacterial siderophore and multidentate iron chelator used to treat iron poisoning, stabilizes HIF-1 (Guo et al., 2016) and helps cardiomyocytes to survive in models of post-ischemic reperfusion in rabbits (Tan et al., 2009). In addition, in various models of *in vivo* ischemia, DFO exhibits neuroprotective effects (Rabinowitz et al., 2010), and DFO is effective in models of Parkinson’s and Alzheimer’s diseases (Ya-Ting et al., 2005). It is important to mention that iron chelators, especially DFO, have a strong capability to inhibit a recently described mode of cell death, ferroptosis, which was implicated in acute and chronic neurodegeneration (Dixon et al., 2012; Hirschhorn and Stockwell, 2019a; Zhang et al., 2020). Similarly, ciclopirox (CPX), a compound that is mainly used in antifungal therapy, is a potent iron chelator and a HIF PHD inhibitor: CPX binds the active site iron with an inhibition constant of 50 nM (Osipyants et al., 2018). Linden et al., using Western blotting, showed that CPX enhances angiogenesis in animal models due to a higher expression of VEGF (Linden et al., 2003). Besides this, CPX affects the functioning of a number of enzymes such as ribonucleotide reductase, cyclin-dependent kinases, and deoxyhypusine hydroxylase. This drug has a relatively low toxicity and holds a potential for inhibiting tumor growth, minimizing diabetic side effects and preventing age-related cardiovascular injury as well as ischemic trauma to neurons.

In addition to HIF PHD inhibition, the compounds of the first two groups can also indirectly affect the replication apparatus of the SARS-CoV-2 virus, as supposed in the review on the use of iron chelators (Liu et al., 2020). Specifically, the nucleotides required for the reverse transcription of viral RNA are synthesized by iron-dependent ribonucleotide reductases, and iron replacement or chelation will prevent viral replication (Romeo et al., 2001).

The third group of inhibitors exploits the fact that HIF PHD belongs to the family of α-ketoglutarate-dependent non-heme iron dioxygenases. Therefore, structural analogs of α-ketoglutarate will work as enzyme inhibitors. The substitution of α-ketoglutarate as a way to inhibit HIF PHD is more specific than iron substitution or chelation since iron is involved in many cellular processes, and the side effects from its displacement or chelation could be deleterious. The human genome contains more than 60 α-ketoglutarate dependent oxygenases, with only some of those being well characterized. Currently, the other best known members of this family include enzymes directly engaged in epigenetic modifications, histone demethylase and cytosine demethylase (TET-enzyme). The inhibition of HIF PHD by substituting α-ketoglutarate might also lead to many off-target effects. The most studied representatives of this group of inhibitors are dimethyloxalylglycine (DMOG) and the compounds developed by FibroGen (FG-4592, or “Roxadustat”) and Akebia (“Vadadustat” (Pergola et al., 2016; Martin et al., 2017)) for the treatment of anemia, inflammation, and ischemia (Haase, 2017; Welden et al., 2017). Vadadustat is currently under clinical trials for the treatment of acute respiratory distress syndrome (ARDS) in COVID-19 patients (Vadadustat for the Prevention and Treatment of Acute Respiratory Distress Syndrome (ARDS) in Hospitalized Patients With Coronavirus Disease 2019 (COVID-19), 2020).

Dimethyl oxalylglycine (DMOG) is one of the most widely used HIF PHD inhibitors in laboratory research. DMOG easily enters the cell, where it is converted to N-oxalyl glycine by the action of carboxyl esterases (Baader et al., 1994; Jaakkola et al., 2001). The incubation of neural cells with 1 mM DMOG for 2 h is sufficient to significantly stabilize HIF isoforms. Acting as an inhibitor of HIF-PHD, DMOG stabilizes HIF and NF-kB transcription factors and suppresses the tumor necrosis factor (Yu et al., 2012). Due to its high structural similarity to α-ketoglutarate, DMOG does not appear to be a specific inhibitor of HIF PHD, and it lowers the activity of most α-ketoglutarate dependent oxygenases. In HEK293T cells, it was also shown that DMOG inhibits histone demethylase JmjC (Hamada et al., 2009). Furthermore, with the given compound’s activity, the activation of the AMPK signal pathway was observed. This pathway allows the cell to adapt to conditions of oxidative stress (Yan et al., 2012). AMPK, like HIF, launches programs that conserve energy, but, in contrast to HIF’s inhibition of mitochondrial function and biogenesis, AMPK enhances mitochondrial growth and activity (Reznick and Shulman, 2006; Winder et al., 2018). However, DMOG may target energy-producing mitochondrial complexes such as α-ketoglutarate dehydrogenase and directly inhibit mitochondrial respiration even before stabilizing HIF (Zhdanov et al., 2015).

Roxadustat (FG-4592) belongs to the group of compounds mimicking the binding mode and therefore, competing with α-ketoglutarate for the HIF-PHD active site. This drug is undergoing clinical trials for the treatment of anemia in patients with chronic kidney insufficiency (Provenzano et al., 2016). Roxadustat, after a 4 weeks-treatment, was shown to downregulate hepcidin (Besarab et al., 2016), whose overproduction is associated with iron-restricted anemia seen in patients with chronic kidney and inflammatory diseases. Furthermore, Roxadustat is neuroprotective and exerts restorative effects in spinal cord injuries (Wu et al., 2016). In fact, Roxadustat has a therapeutic effect in models of Parkinson’s disease (Li et al., 2018). However, despite the major therapeutic advantages of the compound, some side effects were reported in the third phase of clinical trials for patients with chronic kidney insufficiency. Roxadustat successfully passed clinical trials and was approved for the treatment of anemia in China in 2019 (Roxadustat approved in China for the treatment of anemia in non-dialysis-dependent patients with chronic kidney disease, 2019). In 2020 Roxadustat was filed for FDA approval.

Vadadustat, like Roxadustat, successfully passed clinical trials. However, the drug is significantly less characterized with respect to potential applications other than the treatment of anemia. The structural class of Vadadustat and Roxadustat is the same as for collagen prolyl-4-hydroxylase (CP4H) inhibitors which were patented more than 20 years ago (Weidmann et al., 1997). The simple structure of both drugs does not exclude their partial inhibitory activity toward CP4H, the enzyme directly implicated in fibrosis. So, this side effect of the drugs may even be beneficial for COVID-19 patients.

A number of HIF PHD inhibitors of the same class were developed by the other companies. The mode of binding remained essentially the same as for Roxadustat and Vadadustat, but it had a different scaffold for the active site iron binding. Daprodustat (GSK1278863) was developed by GlaxoSmithKline and passed clinical trials (Ariazi et al., 2017). Desidustat or ZYAN1 (Zydus) demonstrated combined effects on endogenous erythropoietin release and efficient iron utilization as well as efficient erythropoiesis and hepcidin downregulation (Jain et al., 2019). In January 2020, Zydus entered into a licensing agreement with China Medical System Holdings for the development and commercialization of Desidustat in Greater China. In September 2020, Japan Tobacco received manufacturing and marketing approval for Enarodustat (ENAROY) in Japan for the treatment of anemia associated with chronic kidney disease. In preclinical research, Enarodustat was shown to be beneficial at the early stages of diabetic kidney disease as well (Hasegawa et al., 2020).

Despite their high activity in inhibiting HIF PHD, α-ketoglutarate analogs may have unpredicted off-target effects due to a big pool of yet to be characterized α-ketoglutarate-dependent enzymes. These off-target effects also stem from the need to use high concentrations of the drugs (orders of magnitude higher than their inhibition constant for HIF PHD) to overcome the competition from 1 to 2 mM intracellular α-ketoglutarate (Thirstrup et al., 2011). The fourth group of HIF PHD inhibitors is represented by 8-oxyquinoline derivatives which bind the active site iron specifically for HIF PHD and not for other known enzymes of this family. The first inhibitor of this group, adaptaquin, was identified 10 years ago in high throughput screening using a cell-based luciferase fusion reporter (HIF1 ODD-luc (Smirnova et al., 2010) (Warshakoon et al., 2006)). Adaptaquin was shown to be neuroprotective in *in vivo* hemorrhagic stroke models, likely working by suppressing the pro-death functions of ATF4 (Aimé et al., 2020), a plausible substrate of HIF PHD isoform 3 (Köditz et al., 2007; Wottawa et al., 2010; Hiwatashi et al., 2011). 8-Oxyquinoline inhibitors of HIF PHD contain a “branched tail” at the 7th position mimicking the fold of HIF peptide at the entry to the active site cavity. Adaptaquin contains one chiral carbon atom and exists as a racemic mixture of two enantiomers that exhibit the same activity in stabilizing HIF (Gaisina et al., 2018). This finding shows that the volume of the HIF PHD active site is sufficient to accommodate more complicated “tails” at the 7th position of the oxyquinoline ring. The optimization effort taken in this laboratory demonstrated that the highest activation effect, in the submicromolar range of concentrations, was observed with *tert*-butyl, isopropyl, or trifluoro-substitution in the *para*-position of the “branched tail” phenyl ring combined with the presence of a methyl group in the *ortho*- or *para*-positions with respect to nitrogen in the “branched tail” pyridine ring as shown in Figure 2 (Poloznikov et al., 2019a). The *tert*-butyl analog of adaptaquin, named neuradapt, was shown to preserve the neuronal network when added at the onset of hypoxia (Savyuk et al., 2020). The testing of both compounds in a liver-on-chip showed no toxicity in up to 100-fold higher concentrations than the biologically active ones (Poloznikov et al., 2019b). However, both adaptaquin and neuradapt are at the stage of pre-clinical investigation and thus, too far from clinical trials. In general, oxyquinolines have a wide spectrum of pharmacological applications (Song et al., 2015; Oliveri and Vecchio, 2016), and the scaffold is well tolerated in humans. Quinine, chloroquine, and hydroxychloroquine also belong to quinolines and have been recommended for the early phase of COVID-19 treatment to prevent viral replication (Li and Cheng, 2020). Later on this recommendation was recalled.

## HIF and SARS-CoV-2 Targets Relationships

The general analyses of the existing investigational drugs targeting HIF PHD allow one to conclude that these drugs are worth trying in COVID-19 patients at the disease stages associated with hypoxia. These drugs launch a number of pro-survival pathways in addition to the classical HIF-pathway, since, as written above, there are multiple client substrates of HIF PHD. However, an integral effect of HIF PHD inhibitors for COVID-19 treatment is difficult to predict. First, there is some uncertainty in the pro-survival mediators stabilized or triggered by the enzyme inhibition, and second, it is due to a great variation of COVID-19 manifestations and post-infection effects. If we focus on the HIF-pathway only, as it is the best studied program launched by a HIF PHD inhibitor, some concerns will arise with respect to the initial steps of viral infection. A hypothesis on HIF activation to treat COVID-19 has been recently put forward (Afsar et al., 2020), however, the literature available on the relationships between HIF and SARS-CoV-2 targets indicates that there could be undesirable effects of HIF activation. Therefore, let us discuss in brief the key steps of SARS-CoV-2 invasion, replication, and triggering the immune response with respect to the known HIF targets.

### Hypoxia and HIF in ACE2 Regulation.

**FIGURE 3:**
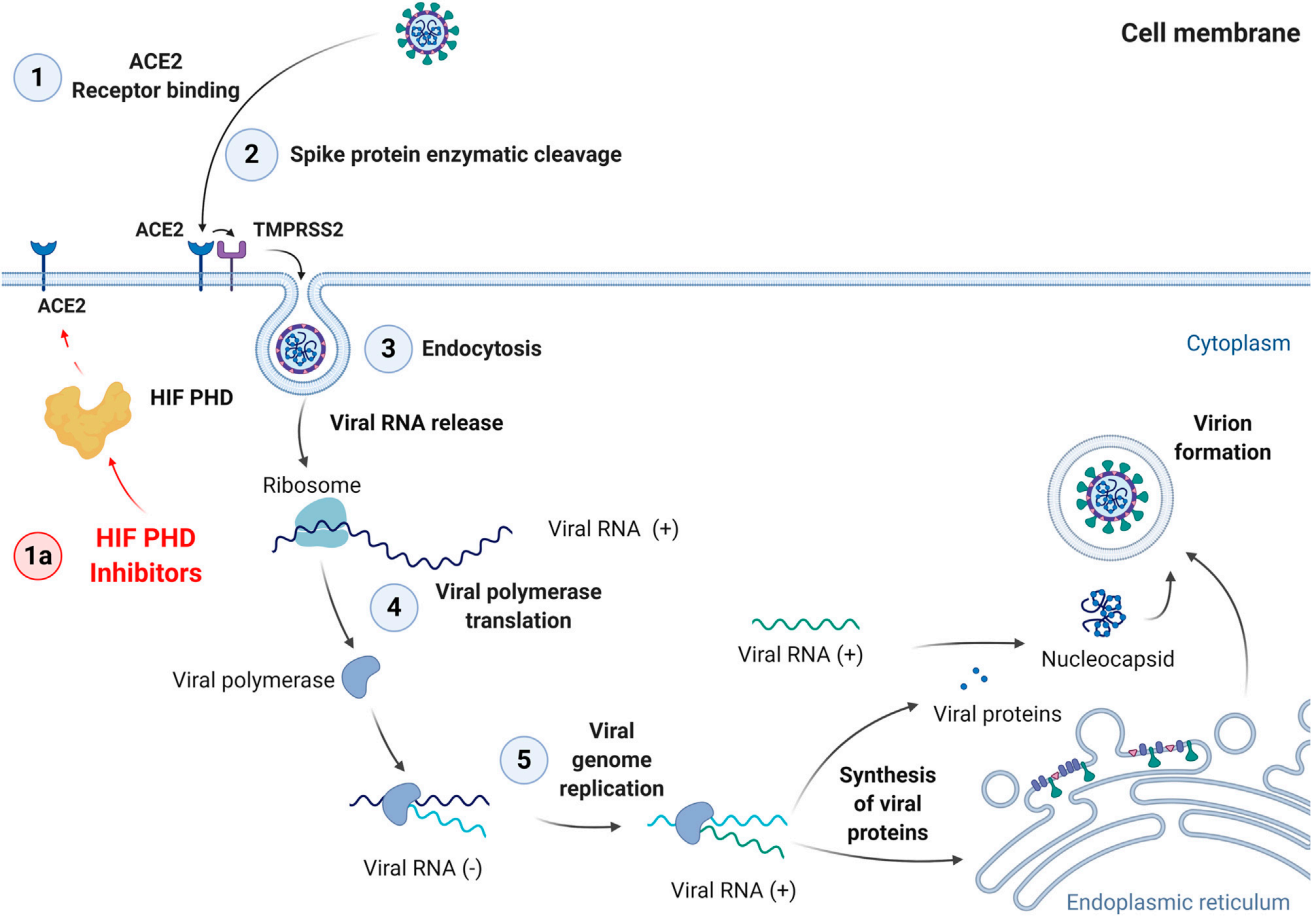
Steps of SARS-Cov-2 cell entry and replication cycle. 1: Binding to ACE2 receptor; 2: Enzymatic cleavage of Spike protein; 3: Endocytosis; 4: Viral polymerase translation; 5: Viral genome replication. The last step is followed by synthesis of viral proteins, virion formation, and exocytosis.

The first step of viral invasion is the interaction of SARS-CoV-2 with the ACE2 (angiotensin converting enzyme 2) receptor on the cell surface (Figure 3).

**FIGURE 4:**
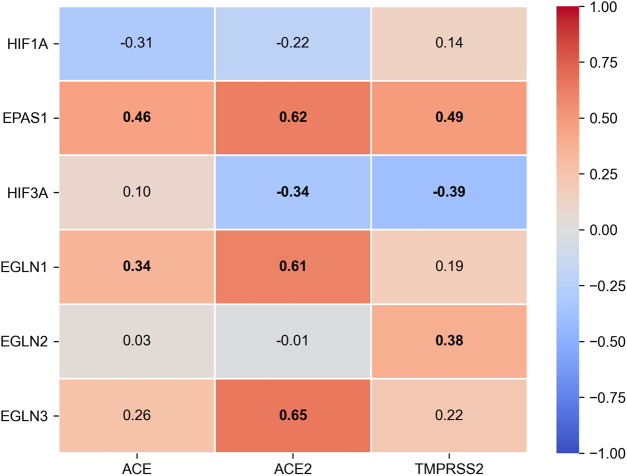
Spearman’s rank correlation matrix between HIF alpha subunits (HIF-1α, HIF-2α encoded by EPAS1, HIF-3α), HIF prolyl-hydroxylase isoforms (EGLN1 encodes PHD2, EGLN2 - PHD1, EGLN3 - PHD3) and ACE2 expression and TMPRSS2 expression in healthy intestinal tissues from TCGA-COAD dataset. RNA sequencing count data for *n* = 38 normal samples were obtained from GDC Data Portal (https://portal.gdc.cancer.gov/) and converted to format of TMM-normalized Fragment Per kilobase of transcript per Million mapped reads (FPKM) with edgeR v3.30.3 (Robinson et al., 2010). Bold labels represent statistically significant correlation (*p* < 0.05).

Despite the fact that the data on the role of ACE2 in health and disease are growing, relatively little is known about the factors controlling its expression, and the available information is often contradictory. Correlations between clinical outcomes and the enzyme expression level on the target cell surfaces of patients from various age groups are of big interest (Cheng et al., 2020). It is assumed that viral entry into the cell is determined by the quantity of the receptors and by the direction of the subsequent intracellular transport of the ACE2-SARS-CoV-2 complex (Magrone et al., 2020). Currently, only scarce data are available on the transcription factors directly regulating the ACE2 promoter. It is known that FOXA2 is essential for basal ACE2 expression, whereas in oxidative stress, the expression is regulated via SIRT1 (Clarke et al., 2014; Pedersen et al., 2017). In the same paper, Clark et al. demonstrated an increased ACE2 expression in hypoxia (Clarke et al., 2014). In contrast, Zhang et al. reported a direct regulation of the ACE receptor by the hypoxia inducible factor (HIF-1), resulting in ACE induction coupled to Ang II accumulation and decreased ACE2 expression (Zhang et al., 2009). The latter observation opens a possibility for the correction of ACE2 expression levels with HIF PHD inhibitors. Interestingly, the analysis of the expression of HIF isoforms in healthy intestinal cells (according to the TCGA database) demonstrates a high level of positive correlation between the isoforms of HIF prolyl hydroxylase, PHD2 (EGLN1) and PHD3 (EGLN3), and ACE2 expression (Figure 4).

This fact supports the hypothesis on a plausible regulation of ACE2 expression through the mechanism of cellular response to hypoxia: an increased expression of HIF PHD leads to a down-regulation of HIF expression in the cell and, subsequently, to an increased expression of ACE2. Another piece of indirect evidence for the role of hypoxia and, accordingly, the role of HIF in ACE2 regulation, is the recently published data that showed people who live at higher altitudes had less serious complications from COVID-19 (Arias-Reyes et al., 2020). Therefore, we can conclude that HIF PHD inhibitors will cause no increase in the ACE2 surface concentration and will not result in attracting more viral particles to the cell surface.

### Hypoxia and HIF in Activation of SARS-CoV-2 Spike Protein

The next step of viral entry is the proteolytic cleavage of the SARS-CoV-2 spike protein, which contains sites for transmembrane serine protease 2 (TMPRSS2) (Zang et al., 2020) and furin. The presence of the latter makes the virus extremely infectious and thus extremely harmful.

TMPRSS2 is a target of the androgen receptor (AR), and this may explain the higher male susceptibility toward this viral infection (Goren et al., 2020; McCoy et al., 2020). HIF1 is necessary but not sufficient for AR transactivation (Mitani et al., 2012). As seen in Figure 4, TMPRSS2 is positively correlated with HIF PHD activity, so the enzyme inhibition will not increase the expression of TMPRSS2 and thus will be harmless with respect to viral entry. Furin has been shown to be a HIF target (McMahon et al., 2005). Very recently, a hypothesis linking the positive effect of hyperoxia in COVID patients with enhanced HIF degradations and, therefore, a reduced expression of furin, was put forward (Koch et al., 2020). With this in mind, one cannot recommend PHD inhibitors at the initial stages of viral infection. Without TMPRSS2 participation, SARS-CoV-2 accesses the cell via an endosomal pathway, in which cathepsin L plays an important role: the spike protein is fusogenically activated by cathepsin L and allows for the fusion of the viral and endosomal membranes. Cathepsin L is a plausible HIF target (Xiaofei et al., 2018) and, as a result, HIF PHD inhibition may increase the expression of cathepsin L via HIF activation.

### Other HIF Targets of Major Concern

There is no consensus on the role of HIF in the modulation of immune response and in counteracting inflammation in severe cases of COVID-19 induced injury. In the recent review, (Del Vecchio and Locatelli, 2020), the authors suggest that HIF and its stabilization by HIF prolyl hydroxylase inhibitors could be beneficial for the treatment of acute lung and kidney injury caused by inflammation. Their conclusion is mostly based on the available literature on pre-treatment with DMOG in animal models. On the other hand, another review on the link between HIF and innate immunity proposes the use of HIF inhibitors for the treatment of the COVID-19 induced inflammatory cascade (Jahani et al., 2020). This viewpoint is supported by the recently published original research paper (Codo et al., 2020) demonstrating that elevated glucose levels and glycolysis promote SARS-CoV-2 replication and cytokine production in monocytes through a HIF-1 dependent pathway resulting in T cell dysfunction and epithelial death.

Recent findings on the urokinase plasminogen activator (uPA)/uPA receptor (uPAR)system suggest its potential role as a main orchestrator of the fatal progression to pulmonary, kidney, and heart failure in coronavirus patients (D’Alonzo et al., 2020). Patients with prolonged background inflammation can exhibit aberrant inflammatory reactions that are well recognized as the main factors potentially resulting in death and are probably sustained by a dysregulated uPA/uPAR system. SuPAR, the soluble form of uPAR, represents a biomarker of disease progression, and its levels correlate well with comorbidities associated with coronavirus patient deaths. There is one recent report on the HIF-mediated upregulation of the uPA receptor during hypoxia (Nishi et al., 2016), and thus the inhibition of PHD may possibly induce the expression of uPAR and contribute to the severity of COVID.

One of the “bad” players in viral and autoimmune diseases is the macrophage migration inhibitory factor (MIF), a pleiotropic proinﬂammatory cytokine that mediates diverse immune responses. MIF counteracts the anti-inﬂammatory effects of glucocorticoids and plays a role in the progress of septic shock. This factor is a well-known HIF target gene and, moreover, MIF and HIF are bound into an autoamplifying feedback loop that can be interrupted by glucocorticoids (dexamethasone in particular) (Gaber et al., 2011). The serum level of MIF is known to be positively correlated with another life-threatening viral infection, Dengue fever (Lai et al., 2020), as MIF is involved in the viral replication of Dengue and in many pathological changes such as vascular leakage. Despite no studies being performed on the link between MIF expression and COVID severity, such a link can be suspected based on the salutary effects of dexamethasone that were recently reported for patients requiring supplemental oxygen (COVID-19 Treatment Guidelines, 2020).

### HIF PHD Inhibitors Versus Recombinant Erythropoietin

Erythropoietin (Epo), the final target of HIF PHD inhibitors that are aimed at replacing Epo IV administration with a tablet, is more than an anti-anemic drug. Epo is known to suppress proinflammatory cytokines, protect cells from apoptosis and promote wound healing. Epo receptors are expressed on a variety of immune cells, enabling Epo to directly modulate their activation, differentiation and function. Right now, researchers are investigating Epo’s ability to ease severe cases of Covid-19 and to protect patients from neurological side effects once the SARS Cov-2 virus attacks the brain. Already, the initial case studies were very positive, and researchers are currently starting a randomized clinical trial to systematically investigate the effects of Epo treatment in COVID-19 patients (see review (Ehrenreich et al., 2020) and refs therein). However, the question is whether Epo administration will be better for COVID patients than treatment with HIF PHD inhibitors, which boost the systemic response. Despite the previously discussed undesirable effects of HIF activation with respect to viral entry, there is another beneficial aspect to HIF PHD inhibition that is not relevant to HIF. Recent studies link the severity of COVID to the so called hyperferritinemia (Vargas-Vargas and Cortés-Rojo, 2020) and thus, to ferroptosis (Hirschhorn and Stockwell, 2019b; Edeas et al., 2020). Interestingly, adaptaquin and its improved variants are potent inhibitors of ferroptosis working via the inhibition of HIF PHD1 (Aimé et al., 2020). Therefore, HIF PHD inhibitors may have additional benefits in comparison to the treatment with Epo alone.

## Conclusion

HIF prolyl hydroxylase inhibitors were recently named among candidate drugs for trials in COVID-19 patients (Del Vecchio and Locatelli, 2020). The authors of this review refer to the available literature on pre-treatment with HIF prolyl hydroxylase inhibitors to ease the various scenarios of hypoxic damage to lungs and kidney. However, clinical evidence at this point shows the benefits of HIF prolyl hydroxylase inhibitors in a post-injury regime only for anemic patients with the compromised ability to synthesize erythropoietin in the kidney or to respond to erythropoietin as we discussed above. Based on the existent knowledge, HIF PHD inhibitors could be beneficial for previously infected patients who still suffer from the consequences of COVID-19. However, at the initial stages of viral replication and disease progression, HIF activation and the induction of HIF targets may increase the proliferation of the virus. Pre-treatment with HIF prolyl hydroxylase inhibitors in COVID-19 may exacerbate the injury, and our conclusions are in agreement with the recently published reviews on the negative role played by HIF in the viral infections in general. We would like to emphasize that HIF is one of more than a dozen substrates for HIF PHD enzymes, and PHD inhibition does not only build up HIF. So, the effect of HIF PHD inhibitors could be different from the effect of hypoxia-stabilized HIF. Still, clinical trials of HIF PHD inhibitors in patients with measurable amounts of the virus must be performed with extreme caution if not avoided altogether.

## Author Contributions

SK, EK, and JM–editing; AAP, DMH, AGT, VIV, SVN, and IGG–concept and writing.

## Funding

The research was funded by Russian Scientific Foundation (Grant No. 20-15-00207).

## Conflict of Interest

The authors declare that the research was conducted in the absence of any commercial or financial relationships that could be construed as a potential conflict of interest.
